# Cell type‐specific signatures of resilience to cognitive decline in the presence of brain pathologies

**DOI:** 10.1002/alz.088929

**Published:** 2025-01-03

**Authors:** Tain Luquez, Dhwani Sreenivas, David A. Bennett, Philip L. De Jager, Vilas Menon

**Affiliations:** ^1^ Columbia University Irving Medical Center, New York, NY USA; ^2^ Columbia University, New York, NY USA; ^3^ Rush University, Chicago, IL USA; ^4^ Department of Neurology, Center for Translational and Computational Neuroimmunology, Columbia University Medical Center, New York, NY USA; ^5^ Center for Translational & Computational Neuroimmunology, Department of Neurology, Columbia University Irving Medical Center, New York, NY USA

## Abstract

**Background:**

To identify discrete and continuous cell type signatures in brain tissue from donors with minimal cognitive decline despite harboring substantial proteinopathies associated with Alzheimer’s Disease and Alzheimer’s Disease‐related dementias.

**Method:**

Three large‐scale single‐nucleus RNA‐seq studies on Alzheimer’s Disease post‐mortem human tissue were re‐annotated and integrated to identify cell type composition associations with cognitive resilience to various neuropathologies. Cell type signatures were defined in two ways: using an integrated clustering approach and using a continuous factor‐based analysis. Cognitive resilience was also defined in two ways: using a categorical grouping of donors as well as a continuous definition based on the slope of decline across each of five cognitive domains.

**Result:**

We report two microglial cell types associated with resilience to Alzheimer’s and alpha‐synuclein proteinopathies. Individuals cognitively resilient to substantial cortical alpha‐synuclein aggregates have higher levels of a microglial subset enriched for redox genes and overexpressing APOE, TYROBP and SPP1. By contrast, cognitively resilient individuals with high amyloid burden show lower proportions of a microglial subset marked by TMEM163+. On the neuronal side, a subset of glutamatergic layer 2/3 neurons was higher in individuals resilient to visuospatial decline despite harboring alpha‐synucleinopathy. A different L2/3 excitatory neuron cluster is depleted in the same individuals, thus highlighting preferential loss or preservation of neuronal subclasses in individuals with cognitive resilience. Finally, we find lower levels of committed oligodendrocyte precursor cells and FTH1+/CRYAB1+ oligodendrocytes in individuals with amyloid and alpha‐synuclein aggregates with better preserved visuospatial, semantic memory and global cognition.

**Conclusion:**

Using a combination of categorical and continuous definitions of resilience, together with existing single‐nucleus RNA‐sequencing data on cortical tissue from >600 human donors, we uncovered cell‐type compositional changes in microglia, neurons and oligodendrocytes that are associated with global and domain‐specific cognitive resilience to amyloid, tau and alpha‐synuclein proteinopathies.

